# Health-Related Quality of Life in Chemotherapy Patients Using the European Organization for Research and Treatment of Cancer Quality of Life Questionnaire-Core 30 (EORTC QLQ-C30): A Single-Institution-Based Study From Lahore, Pakistan

**DOI:** 10.7759/cureus.86627

**Published:** 2025-06-23

**Authors:** Jawayria Sajid, Kinza Sabir, Nauman Ismat Butt, Barak Waris, Imania Khizar, Ayman Bashir

**Affiliations:** 1 Medical Oncology, Shalamar Hospital, Shalamar Medical and Dental College, Lahore, PAK; 2 Medical Oncology, Nawaz Sharif Social Security Teaching Hospital, Lahore, PAK; 3 Internal Medicine, Chaudhry Muhammad Akram Teaching and Research Hospital, Azra Naheed Medical College - Superior University, Lahore, PAK

**Keywords:** cancer, carcinoma, chemotherapy, eortc qlq-c30 questionnaire, european organization for research and treatment of cancer quality of life questionnaire (eortc qlq-c30), malignancy, oncology, quality of life (qol)

## Abstract

Objective

The objective of this study was to assess health-related quality of life (QoL) in patients receiving injectable chemotherapy using the European Organization for Research and Treatment of Cancer Quality of Life Questionnaire-Core 30 (EORTC QLQ-C30) at the Oncology Department of a teaching hospital in Lahore, Pakistan.

Methods

This cross-sectional observational study was carried out at the Department of Medical Oncology, Nawaz Sharif Social Security Teaching Hospital, Lahore, Pakistan, between January and April 2025. A total of 150 patients receiving injectable chemotherapy were enrolled using a non-probability consecutive sampling method. Inclusion criteria required participants to be 18 years or older, of either gender, with a confirmed cancer diagnosis of any stage for at least three months, a life expectancy exceeding six months, and the ability to provide informed consent. Life expectancy exceeding six months was estimated by the treating oncologist based on clinical judgment, considering cancer type and stage, performance status, treatment response, and overall health condition. Patients were excluded if they were undergoing radiotherapy; were bedbound, disabled, or too unwell to participate as judged by the clinical team; had coexisting chronic conditions such as chronic liver disease, chronic kidney disease, or congestive heart failure; had psychiatric comorbidities; or declined to participate. The EORTC QLQ-C30 (v3.0) was used to assess health-related QoL. Following institutional ethical approval and written informed consent, demographic and clinical data, including age, gender, cancer type, presence and location of metastases, and number of injectable chemotherapy cycles, were collected. Data analysis was performed using Statistical Product and Service Solutions (SPSS, v23; IBM SPSS Statistics for Windows, Armonk, NY). Group comparisons were conducted using independent t-tests and one-way analysis of variance (ANOVA), with a p-value ≤0.05 considered statistically significant.

Results

The mean age of the patients was 52.7±12.5 years, with 114 (6.0%) females. The most common malignancy was breast carcinoma, seen in 74 (49.3%) patients, followed by hematological malignancies (21, 14.0%) and periampullary cancer (17, 11.3%). Metastatic disease was reported in 42 patients (28.0%), with 24 (10.0%) having bone metastasis. Seventy-four (49.4%) had received three to four injectable chemotherapy cycles, whereas 62 (41.3%) had received ≥5 injectable chemotherapy cycles at the time of assessment. Age was notably linked to multiple domains of EORTC QLQ-C30, including physical functioning (p=0.017), social functioning (p=0.006), nausea/vomiting (p=0.029), appetite loss (p=0.035), constipation (p=0.040), and financial difficulties (p=0.045). The presence of metastasis significantly affected physical functioning (p=0.037), fatigue (p=0.025), pain (p=0.019), and financial difficulties (p=0.022), while the type of cancer showed a significant association with diarrhea scores (p=0.015). No significant association of gender and number of chemotherapy cycles was seen with QoL parameters.

Conclusion

This study highlights that health-related QoL in chemotherapy patients was significantly influenced by age, presence of metastasis, and type of cancer. These findings underscore the importance of individualized supportive care strategies, particularly for older patients and those with advanced disease, to improve their overall well-being.

## Introduction

A major health issue affecting the physical, psychological, and spiritual well-being of patients, cancer is the second leading cause of death globally, responsible for about one in six deaths [1]. Approximately 70% of these cancer-related deaths occur in middle- and low-income countries [2]. In addition to mortality, cancer not only causes significant morbidity but also impacts quality of life (QoL), including health, social interaction, and overall function [3,4]. According to the World Health Organization (WHO), QoL is an individual’s view of their life within their cultural and value context, influenced by expectations and personal goals [5]. A key focus in current healthcare, especially for chronic illnesses, QoL is a vital part of cancer care because both the illness and its treatment, including chemotherapy, can affect patients’ well-being and reduce QoL [1,3]. Therefore, evaluating QoL helps to address these patients’ emotional, physical, and social needs. Pruthi et al. reported that emotional well-being declined after chemotherapy [6]. Mason et al. reported that 39% of cancer patients experienced psychological distress, more so among women and the elderly [7].

Effective cancer control requires timely detection, prompt treatment, symptom relief, and support. However, middle- and low-income countries such as Pakistan face numerous challenges, including limited access to screening, delayed diagnosis, poor treatment options, and lack of pain relief and psychosocial support [6,7]. This results in a significant impact on the QoL of cancer patients in addition to causing morbidity and mortality. Despite the high disease burden, QoL remains understudied in many regions. Cancer patients often experience poor quality of life due to limited awareness, delayed diagnoses, inadequate care, workforce shortages, financial barriers, high treatment costs, lack of insurance, and the physical and emotional burden of cancer symptoms and treatment side effects, underscoring the urgent need for coordinated, patient-centered care [8,9]. Despite global attention to cancer care, a lack of context-specific data on QoL is seen in middle- and low-income countries, especially Pakistan. Therefore, we designed the present study to evaluate the QoL of adult cancer patients receiving chemotherapy in Lahore, Pakistan, using the validated European Organization for Research and Treatment of Cancer Quality of Life Questionnaire-Core 30 (EORTC QLQ-C30).

## Materials and methods

This observational cross-sectional study was conducted at the Department of Medical Oncology, Nawaz Sharif Social Security Teaching Hospital, Lahore, Pakistan, from January to April 2025. A total of 150 patients undergoing injectable chemotherapy were recruited using a non-probability consecutive sampling technique. The sample size was based on the feasibility of recruiting patients during the study duration and prior studies assessing QoL among cancer patients using similar tools. Eligible participants were adults aged ≥18 years of both genders, with a confirmed diagnosis of cancer of any stage for at least 3 months, a life expectancy of more than 6 months and the ability to provide informed consent. Life expectancy exceeding six months was estimated by the treating oncologist based on clinical judgment, considering cancer type and stage, performance status, treatment response and overall health condition. Patients receiving radiotherapy; those who were bedbound, disabled or too ill to participate as judged by the clinical team; those who had co-existing chronic illnesses such as chronic liver disease, chronic kidney disease or congestive cardiac failure; psychiatric comorbidities; and those unwilling to participate were excluded.

Developed by the European Organisation for Research and Treatment of Cancer (EORTC), the EORTC QLQ-C30 (Version 3.0) questionnaire was used to assess health-related quality of life. The EORTC QLQ-C30 includes 30 items grouped as, five functional scales: physical, role, cognitive, emotional and social functioning; three symptom scales: fatigue, pain, and nausea/vomiting; single items addressing symptoms of dyspnea, appetite loss, insomnia, constipation, diarrhea; financial difficulties; and a global health status/QoL scale [10,11]. Each scale and single item on the EORTC QLQ-C30 is linearly transformed and scored from 0 to 100. For the functional scales, a higher score reflects better functioning. Conversely, for the symptom scales and individual symptom items, higher scores indicate greater severity or burden. The Global Health Status/QoL scale also ranges from 0 to 100, where higher scores represent better perceived overall health and quality of life. The EORTC QLQ-C30 questionnaire is available online (https://qol.eortc.org/questionnaire/eortc-qlq-c30/).

After obtaining institutional ethical approval and informed consent, demographic and clinical data, including age, gender, type of cancer, presence and site of metastasis, and number of injectable chemotherapy cycles, were recorded. Patients were classified as having metastatic disease if they had radiologically or histologically confirmed distant metastasis, including both unifocal and multifocal involvement. Regional nodal spread was also included as metastatic if confirmed beyond locoregional boundaries, based on tumor type. Data were analyzed using Statistical Product and Service Solutions (SPSS, version 23.0; IBM SPSS Statistics for Windows, Armonk, NY). Descriptive statistics were used for demographic and clinical variables. Means and standard deviations were calculated for continuous variables; frequencies and percentages for categorical variables. Group comparisons were made using t-tests for binary variables, and analysis of variance (ANOVA) for variables with more than two categories. A p-value of ≤0.05 was considered statistically significant.

## Results

Of the 150 injectable chemotherapy patients included in the study, 114 (6.0%) were female. The mean age is 52.7±12.5 years, with 79 (52.6%) patients aged ≥54 years. The most common malignancy was breast carcinoma, seen in 74 (49.3%) patients, followed by hematological malignancies (21, 14.0%) and periampullary cancer (17, 11.3%), as shown in Table 1. Metastatic disease was present in 42 patients (28.0%), including both unifocal and multifocal metastasis. The most common site was bone metastasis (24, 10.0%). As depicted in Table 1, 74 (49.4%) had received three to four injectable chemotherapy cycles, whereas 62 (41.3%) had received ≥5 injectable chemotherapy cycles at the time of assessment.

**Table 1:** Demographic and Clinical Variables (n=150). * Frequencies of metastatic sites are not mutually exclusive; patients with multifocal disease are represented in more than one category.

Demographic and Clinical Variables		Frequency n(%)
Age	≤53 years	71 (47.4%)
	≥54 years	79 (52.6%)
Gender	Female	114 (76.0%)
	Male	36 (24.0%)
Type of cancer	Breast carcinoma	74 (49.3%)
	Hematological malignancies	21 (14.0%)
	Periampullary cancer	17 (11.3%)
	Ovarian carcinoma	12 (8.0%)
	Cervical malignancy	10 (6.7%)
	Lung carcinoma	09 (6.0%)
	Colorectal carcinoma	07 (4.7%)
Metastasis	Present	42 (28.0%)
	Absent	108 (72.0%)
Site of metastasis*	Bone metastasis	24 (10.0%)
	Nodal metastasis	13 (9.3%)
	Liver metastasis	12 (8.0%)
	Lung metastasis	12 (8.0%)
	Peritoneal metastasis	02 (1.3%)
Number of chemotherapy cycles	≤2	14 (9.3%)
	3-4	74 (49.4%)
	≥5	62 (41.3%)

Table 2 presents mean ± standard deviation (SD) scores for each QLQ-C30 domain, along with p-values from ANOVA or t-tests evaluating associations with age, gender, type of cancer, presence of metastasis, and number of injectable chemotherapy cycles. Age was notably linked to multiple domains, including physical functioning (p=0.017), social functioning (p=0.006), nausea/vomiting (p=0.029), appetite loss (p=0.035), constipation (p=0.040), and financial difficulties (p=0.045). Additionally, the presence of metastasis significantly affected physical functioning (p=0.037), fatigue (p=0.025), pain (p=0.019), and financial difficulties (p=0.022), while the type of cancer showed a significant association with diarrhea scores (p=0.015), as demonstrated in Table 2. No significant association of gender and number of chemotherapy cycles was seen with QoL parameters.

**Table 2:** Association of demographic and clinical variables with the European Organization for Research and Treatment of Cancer Quality of Life Questionnaire-Core 30 (EORTC QLQ-C30) (n=150). ^a ^EORTC QLQ-C30: European Organization for Research and Treatment of Cancer Quality of Life Questionnaire-Core 30; ^b ^QoL: Quality of Life; ^* ^p-value ≤0.05 significant using t-tests (age, gender, and metastasis) and ANOVA (type of cancer and number of chemotherapy cycles).

EORTC QLQ-C30^a^	Mean ± SD	p-value*				
		Age	Gender	Type of Cancer	Metastasis	Number of Cycles
Physical Functioning	30.2 ± 26.2	0.017*	0.470	0.406	0.037*	0.635
Role Functioning	39.5 ± 33.5	0.154	0.530	0.063	0.155	0.300
Emotional Functioning	30.8 ± 26.3	0.329	0.410	0.285	0.299	0.490
Cognitive Functioning	23.1 ± 25.2	0.659	0.994	0.207	0.882	0.421
Social Functioning	27.3 ± 30.2	0.006*	0.508	0.296	0.629	0.611
Fatigue	57.6 ± 24.5	0.185	0.659	0.246	0.025*	0.107
Pain	40.0 ± 30.6	0.294	0.641	0.189	0.019*	0.243
Nausea/Vomiting	36.2 ± 35.8	0.029*	0.698	0.103	0.583	0.680
Dyspnea	19.5 ± 29.5	0.627	0.894	0.143	0.441	0.396
Insomnia	34.2 ± 36.7	0.410	0.066	0.097	0.304	0.231
Appetite Loss	31.5 ± 33.2	0.035*	0.414	0.212	0.321	0.738
Constipation	19.5 ± 30.5	0.040*	0.870	0.638	0.929	0.105
Diarrhea	24.2 ± 36.0	0.351	0.149	0.015*	0.742	0.222
Financial Difficulties	28.4 ± 37.4	0.045*	0.422	0.726	0.022*	0.209
Global Health/QoL^b^	60.5 ± 27.2	0.172	0.635	0.072	0.963	0.388

## Discussion

In the present study, the mean Global Health/QoL score was 60.5, which is considerably lower than the 81.4 reported in a study from Sweden [12]. However, it was higher than the QoL scores reported in studies from middle- and low-resource countries, which found mean scores of 35.7 and 44.3, respectively [8,13]. Our findings were comparable to a study conducted in Ethiopia, which reported a mean QoL score of 52.7 [14]. These variations in QoL score may be attributed to differences in healthcare infrastructure, availability of supportive care services, socioeconomic conditions, and cultural perceptions of illness and well-being. The present study found no significant relationship between the mean global health/QoL score and factors, such as age, gender, cancer type, presence of metastasis, or number of chemotherapy cycles. These findings are consistent with those of Dehkordi et al., who observed no significant link between QoL and demographics or disease severity, including age, gender, social status, marital status, occupation, and disease stage [15]. Comparable results have also been reported in other studies [16,17].

In the current study, role functioning showed the highest functional score (mean: 39.5), whereas cognitive functioning was the lowest (mean: 23.1). Age was significantly associated with physical functioning (p=0.017) and social functioning (p=0.006) in our study, while the presence of metastasis had a notable impact on physical functioning (p=0.037). Conversely, an Ethiopian study identified role functioning as the lowest and social functioning as the highest scoring domain [18]. Another study reported emotional functioning as the most preserved domain, with a mean score of 61.0 [14]. In a study from Punjab, India, none of the functional scales, including physical, role, emotional, cognitive, or social functioning, showed significant changes [19]. In a separate study from Uttarakhand, India, Ramasubbu et al. similarly observed that functional well-being was the most negatively affected, with a mean score of 13.9 [1]. These findings align with previous evidence highlighting autonomy as a core element of quality of life in chronic illness [20]. Moreover, chemotherapy is known to reduce physical capacity and exacerbate fatigue in cancer patients and survivors [21]. These differences in functional domain scores across studies may be explained by the variations in cultural factors, healthcare access, and differences in treatment protocols.

Among the symptom scales, fatigue had the highest mean score at 57.6, while dyspnea and constipation scored the lowest at 19.5 each. This contrasts with other studies where dyspnea was reported as the most severe symptom [12,22]. Age was significantly associated with nausea/vomiting (p=0.029), appetite loss (p=0.035), and constipation (p=0.040) in our study, while the presence of metastasis had a notable impact on fatigue (p=0.025) and pain (p=0.019). Diarrhea, with a mean score of 24.2 in our study, was also one of the least reported symptoms, consistent with findings from other research [12,23]. The type of cancer was significantly associated with diarrhea scores (p=0.015) in our study. The mean score for financial impact in the present study was 28.4, which is considerably lower than the financial difficulty scores of 82.6 and 54.1 reported in studies from Ethiopia and Vietnam, respectively [12,24]. In our study, age (p=0.045) and presence of metastasis (p=0.022) had notable impacts on financial difficulties. Singh et al. reported a significant rise in financial burden, driven by the high cost of medications and loss of income during repeated chemotherapy sessions [19]. These differences may result from variations in patient characteristics, including patient age, sample size, and cancer type; the toxicity of chemotherapy agents; healthcare costs and insurance; availability of financial support; and cultural differences in symptom perception and reporting.

The present study has certain limitations that should be considered when interpreting the results. It was conducted at a single institution and had a relatively small sample size, which may limit the generalizability of the findings to other settings or regions, reduce statistical power, and restrict the ability to detect less prominent associations. The cross-sectional design of our study captures QoL at a single point in time, preventing conclusions regarding changes over the course of treatment. Reliance on self-reported data may cause recall or response bias. Finally, various potential confounding variables, including socioeconomic status, education, and employment, type of specific chemotherapeutic drug being given, and psychological support systems, were not fully controlled for, which might have influenced the results. Future studies should include larger, multi-center research with longitudinal designs to better understand the evolving QoL in cancer patients on chemotherapy and identify effective interventions to improve patient outcomes.

## Conclusions

This study highlights the significant influence of demographic and clinical factors on QoL among chemotherapy patients, as measured by the EORTC QLQ-C30. Older patients and those with metastatic disease reported greater impairments in physical and social functioning, as well as higher symptom burden and financial distress. In contrast, gender and the number of chemotherapy cycles did not significantly affect any QoL parameter. These findings underscore the importance of individualized supportive care strategies, particularly for older patients and those with advanced disease, to improve their overall well-being during chemotherapy.
